# Effects of soil properties on heavy metal bioavailability and accumulation in crop grains under different farmland use patterns

**DOI:** 10.1038/s41598-022-13140-1

**Published:** 2022-06-02

**Authors:** Decong Xu, Zhangjun Shen, Changming Dou, Zhiyong Dou, Yang Li, Yi Gao, Qingye Sun

**Affiliations:** 1 School of Life Science, Hefei Normal University, Hefei, 230061 China; 2 School of Resources and Environmental Engineering, Anhui University, Hefei, 230061 China; 3 Anhui Academy of Environmental Sciences, Hefei, 230061 China

**Keywords:** Ecology, Environmental sciences

## Abstract

Mining activities have increased the accumulation of heavy metals in farmland soil and in food crops. To identify the key soil properties influencing heavy metal bioavailability and accumulation in food crops, 81 crop samples and 81 corresponding agricultural soil samples were collected from rape, wheat, and paddy fields. Heavy metal (copper (Cu), zinc (Zn), lead (Pb), cadmium (Cd), iron (Fe), and manganese (Mn)) concentrations in soils and rape, wheat, rice grains were determined using inductively coupled plasma atomic emission spectroscopy, and soil physicochemical properties (pH, organic matter, total nitrogen, total phosphorus, available phosphorus, and available potassium (AK)) were analyzed. Soil extractable metals were extracted using various single extractants (DTPA, EDTA, NH_4_OAc, NH_4_NO_3_, and HCl). The average concentrations of Cu, Zn, Pb, Cd, and Mn in the soil samples all exceeded the local geochemical background value (background values of Cu, Zn, Pb, Cd, and Mn are 43.0, 81.0, 28.5, 0.196, and 616 mg/kg, respectively), and Cd over-standard rate was the highest, at 98%. Furthermore, soil total Cd concentrations (0.1–24.8 mg/kg) of more than 86% of the samples exceeded the soil pollution risk screening value (GB 15618-2018). The sources of Cu, Zn, Pb, Cd, and Mn in soils were mainly associated with mining activities. The key factors influencing heavy metal bioavailability were associated with the types of extractants (complexing agents or neutral salt extractants) and the metals. Cd and Pb concentrations in most wheat and rice grain samples exceeded the maximum allowable Cd and Pb levels in food, respectively, and Cd concentrations in approximately 10% of the rice grain samples exceeded 1.0 mg/kg. Furthermore, rice and wheat grains exhibited higher Cd accumulation capacity than rape grains, and despite the high soil Cd concentrations in the rape fields, the rape grains were safe for consumption. High soil pH and AK restricted Cd and Cu accumulation in wheat grains, respectively. Soil properties seemed to influence heavy metal accumulation in rice grains the most.

## Introduction

Mining activities have led to heavy metal contamination in most agricultural soils and in food crops^1–4^. Most of the heavy metals (copper (Cu), zinc (Zn), manganese (Mn) etc.) are essential nutrients required for crop growth at low levels^5^. However, other metals (cadmium (Cd), lead (Pb), arsenic (As), mercury etc.) can also be absorbed and accumulated in crops, although they are not essential for crop growth^6–8^. Therefore, heavy metal contamination of agricultural soils and food crops near mining sites have raised environmental and food safety concerns globally. Metal accumulation in crops occurs mainly via absorption by roots from contaminated soils, and the amounts accumulated vary across crop species and soil heavy metal bioavailability^9–11^.

Currently, soil heavy metal bioavailability is generally expressed based on the relationship between the amounts of heavy metals absorbed by plant tissues and heavy metal concentrations in soil, and soil heavy metal concentrations are evaluated following extraction by various methods. Among the common extraction methods, single extraction methods (diethylenetriaminepentaacetic acid (DTPA), ethylenediaminetetraacetic acid (EDTA), hydrochloric acid (HCl) extraction methods, etc.) are the most extensively used methods, considering their relative simplicity and low time requirements. Nevertheless, numerous studies investigating soil heavy metal bioavailability have applied either single or mixed extractants^12,13^, and no single method has been applied universally. Most studies have demonstrated that the amounts of heavy metal in soil determined by various extraction methods are only suitable for predicting bioaccumulation of specific metals in certain plants^14–16^. The reason is that soil properties^17^ and agronomic management practices^18–20^ can influence heavy metal bioavailability. Therefore, to investigate soil heavy metal bioavailability and accumulation in food crops, several researchers have attempted to establish empirical models to explain how soil extractable heavy metal concentrations and other soil properties influence heavy metal concentrations in crop tissues^14,21,22^. However, on account of the various designs and experimental conditions, comprehensive information with regard to the key soil properties influencing soil heavy metal bioavailability and metal bioaccumulation in food crops is lacking, especially under different farmland use patterns, which have been reported to influence soil heavy metal bioavailability and metal bioaccumulation in food crops^23–25^.

Tongling city, in Anhui Province, China, is known as the "Copper capital of ancient China, contemporary copper mining base." The area has been mined mainly for Cu and iron (Fe) ores since the Shang and Zhou dynasties, and the activities have been carried out for more than 3500 years^26^. In the region, soils, water, and sediment are contaminated mainly by Cd, Cu, As, and Zn^8,27^. The aim of the present study was to provide a reference for determining the appropriate tillage practices for the production of crops with low heavy metal concentrations. The specific objectives were to (1) determine key soil chemical properties (pH, soil organic matter (SOM), total nitrogen (TN), total phosphorus (TP), available phosphorus (AP), and available potassium (AK)) in soils obtained from rape fields, wheat fields, and paddy fields, which represent major farmland use activities in the Tongling mining area; (2) investigate Cu, Zn, Pb, Cd, Fe, and Mn concentrations in the rape, wheat, and rice grains, and total and extractable metal concentrations in soils; (3) identify spatial distribution characteristics and sources of soil heavy metals in the study area; (4) reveal the key factors influencing heavy metal bioavailability and accumulation in different crop species.

## Materials and methods

### Statements

A statement specifying the appropriate permissions for collection of plants: the collection of plant materials (rape (*Brassica juncea*), wheat (*Triticum aestivum*), and rice (*Oryza sativa*) grains) in this study complied with the Chinese government (Ministry of Ecology and Environment of the People’s of China) guidelines and legislation. (Studies complied with local and national regulations for this research was funded by National Natural Science Foundation of China (41171418)).

### Soil and crop sampling

**Figure 1:**
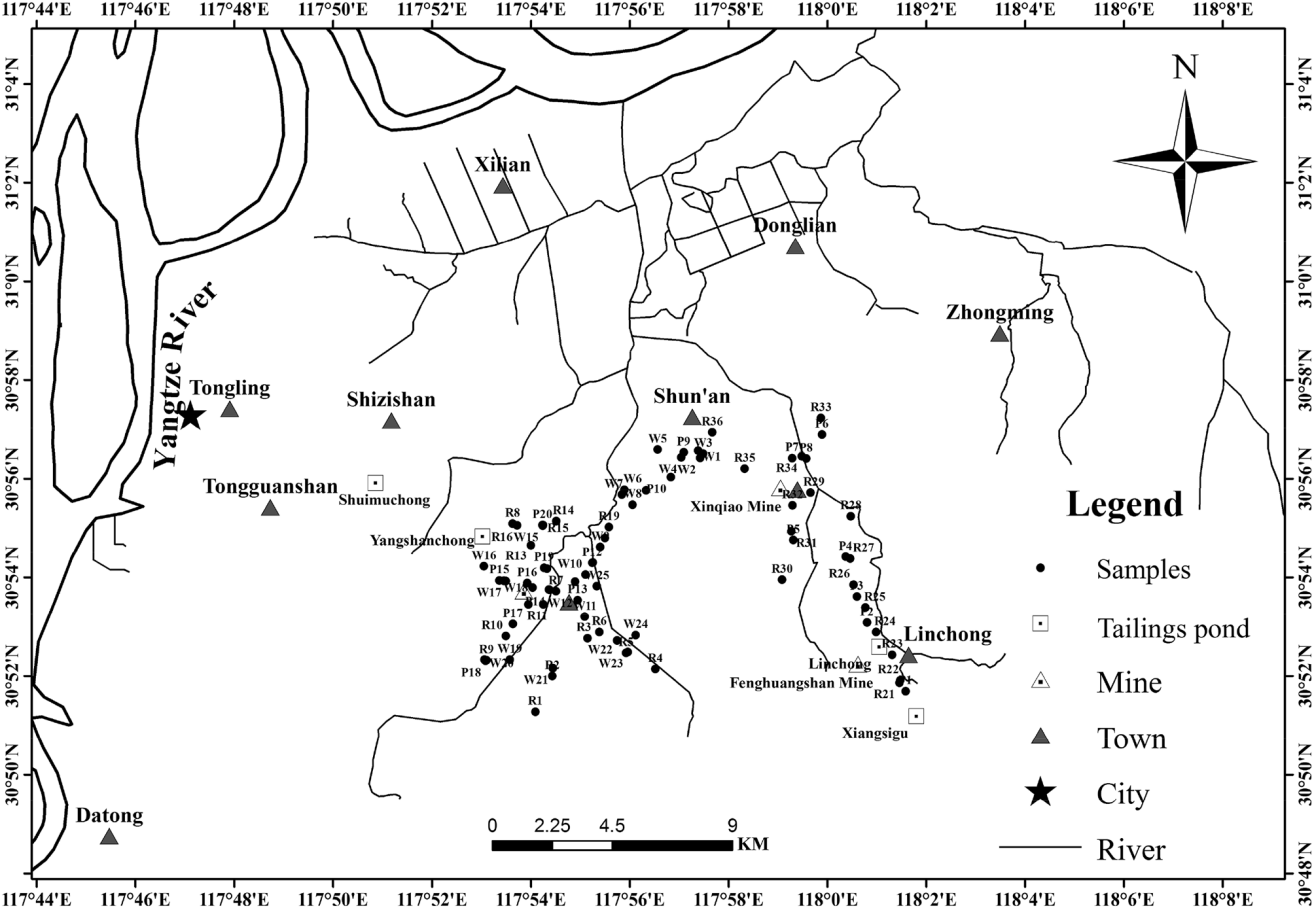
Location map of the study area and topsoil samples.

Figure 1 displays the locations of the sampling sites. Tongling mining area has a humid subtropical climate, and the average temperature and rainfall are 16.2 °C and 1346 mm, respectively. Most of the soils are brown–red soil (Ferralosols) and the mineral deposits are iron–copper–gold–sulfide deposits^8^. The lithological setting of the investigated area was as described by Xu et al.^8^. The natural background values of Cu, Zn, Pb, Cd, and Mn in Tongling are 43.0, 81.0, 28.5, 0.196, and 616 mg/kg, respectively^26^.

A handheld GPS device was used to determine the locations of the sampling sites and all sampled fields are located in the same lithological unit. Furthermore, only fields in which the same crop was planted each year were considered for sampling. Soil samples (2.5 kg) were obtained from a depth of 0–20 cm at 81 sites, including 36, 25, and 20 soil samples from the rape fields (R), wheat fields (W), and paddy fields (P), respectively. Each soil sample was composed of five sub-samples, and each of the sub-samples were separated by at least 20 m^8^. In addition, 81 crop samples, including rape (*Brassica juncea*), wheat (*Triticum aestivum*), and rice (*Oryza sativa*) grains were collected from the corresponding soil sampling sites. At each sampling site, 10 replicate crop samples were mixed to form a composite sample. All the soil samples were air-dried, ground, and homogenized, and then passed through a 2-mm mesh sieve for soil pH, AP, AK, and extractable metal concentration determination. For total heavy metal, TP, TN, and SOM concentration determination, a part of each sample (approximately 1.25 kg) was ground again and passed through a 0.15-mm mesh sieve.

All the grains were cleaned with running water and deionized water, and then dried at 105 ℃ for 30 min for fixation, and then dried at 70 ℃ until constant weight. Dried grain samples were ground, homogenized, and passed through a 0.15 mm mesh sieve, and then stored in a desiccator in polyethylene zip-bags for later heavy metal determination analyses.

### Sample analysis

All chemicals used for metal concentration analysis were guarantee reagents, and analytical reagents were used for soil physicochemical property analyses. Soil pH was measured in water^8^. Organic matter content was determined using the K_2_Cr_2_O_7_–H_2_SO_4_ oxidation method, according to Xu et al.^8^. Soil TN was determined using the Kjeldahl method, according to Zhan and Sun^28^. Soil samples were digested using nitric perchloric acid, and then TP was determined using the molybdenum-blue colorimetric method. Soil AP was determined using the phosphomolybdate blue colorimetric method, according to Zhan and Sun^28^. Soil AK was determined using the ammonium acetate lixiviation method^29^.

To prepare samples for total metal concentration analysis, soil and crop samples were digested in a Microwave Digestion System (Speedwave-4, Berghof)^8^. Sieved soil samples were weighed (0.5 g) and placed into 50-mL polyfluorotetraethylene tubes. Subsequently, 8 mL concentrated nitric acid (HNO_3_) and 2 mL concentrated hydrogen fluoride were added, and the mixture digested. When the digestion reaction was complete, the digest solution was transferred and heated until it reduced to about 5 mL. Subsequently, 2 mL perchloric acid was added and heated again until it reduced to about 2 mL. For crop samples, similar to in the method of preparation of soil samples, 0.25 g of dried sample was digested with 5 mL HNO_3_ and hydrogen peroxide (5:1 ratio). The soil and crop digests were diluted to 25 mL with deionized water. Blank samples were also prepared, and 30% of parallel samples were taken from individuals randomly. Soil extractable metals were extracted using DTPA, EDTA (pH = 7.0), NH_4_OAc (pH = 7.0), NH_4_NO_3_, and HCl, as described by Xu et al.^8^. Metal concentrations in crops were analyzed by high-resolution inductively coupled mass spectrometry (Element II, Waltham, MA, USA). Total and extractable concentrations of metals in soils were determined using inductively coupled plasma atomic emission spectroscopy (IRIS Intrepid II XPS, Waltham, MA, USA). The blank samples were used for the correction of measured signals. When the detection limit was determined, the blank was repeated 10 times, and the triple standard deviation of the determination result was taken as the limit of detection of each element. Each determination was performed in triplicate for each sample, and the quality of metal determination was ascertained based on blanks and certified reference materials, once every 10 samples. Standard soil and crop reference materials (soil (GB (government standard) W07429), wheat (GBW10046), and rice (GBW10045)) were used in the metal analyses. All the recoveries of the metals in the soil and plant reference materials were approximately 88–111%.

### Data analysis

Bioaccumulation factors (*BAF*) is a common method of estimating crop capacity to absorb and transfer metals from soil. *BAF* was calculated as follows:

1 $$BAF = C_{{{\text{plant}}}} /C_{{{\text{soil}}}}$$

where *C*_*plant*_ is the metal concentration in the grains and *C*_*soil*_ represents the total concentration of the metals in the soil^30^. Availability index (AI) is the ratio of extractable concentration of a metal to the total concentration in soil.

### Statistical analysis

Pearson correlation, regression analysis, and principal component analysis (PCA) were performed using IBM SPSS Statistics 19.0 (IBM Corp., Armonk, NY, USA). The spatial distribution map of soil heavy metal concentrations was generated using ArcGIS10.2 (Esri, Redlands, CA, USA). Pearson correlation coefficients were used to evaluate the correlations between soil properties and extractable metal concentrations, and metal concentrations in soil and crop grains. Stepwise multiple linear regression models including soil properties (independent variables) and extractable metals (dependent variables), and models including soil properties and metal concentrations (independent variables) and metal concentrations in crop grains (dependent variables) were both established. The *BAF* values and metal concentrations in crop grains and soils associated with the different farmland use patterns were compared using multiple comparisons. The level of significance was defined as *p* < 0.05. To facilitate correlation analysis, the metal concentrations below the detection limits (detection limits for Cu, Zn, Pb, Cd, Fe, and Mn were 0.005, 0.005, 0.03, 0.002, 0.002, and 0.0005 mg L^−1^, respectively) were replaced with a half of the detection limit value, and this could not affect the conclusions. Furthermore, to guarantee normal distribution, the SOM, TN, TP, AP, and AK, and metal concentrations in soil and crop grains were Log_10_-transformed prior to correlation and regression analyses.

## Results

### Soil physicochemical properties

Table 1 shows the basic chemical characteristics of the 81 soil samples. The pH values of most of the soil samples from the rape fields and the paddy fields ranged between 6.5 and 7.5, while the pH values of most of the soil samples from the wheat fields were less than 6.0. The relatively low pH values for soils from the wheat fields could be due to traditional farming practices adopted in the farms, including continuous cropping. In addition, acidic soil is conducive for wheat growth. Organic matter contents were generally low in all the soils, and soils from the rape fields had the lowest organic matter contents. Generally, the mean soil total N contents in the fields were in the order of rape fields (756 mg/kg) < wheat fields (973 mg/kg) < paddy fields (1034 mg/kg), while the mean soil available K contents in the fields were in the order of paddy fields < wheat fields < rape fields. The mean soil total P and available P contents both exhibited the order of wheat fields < paddy fields < rape fields. Considering the relatively low SOM, TN, TP, AP, and AK contents, the studied soils should be fertilized regularly. Furthermore, the considerable variations in the parameters (pH, SOM, TN, TP, AP, and AK) could be a attributed to the varied farming practices of small-holder farmers, which are suitable for studying how soil properties influence heavy metal bioavailability and accumulation in different soil-cropping systems.

**Table 1:** Summary of main chemical properties of soils collected in the study area.

Parameter	Item	R (n = 36)	W (n = 25)	P (n = 20)
pH (H_2_O)	Mean ± SD	6.7 ± 0.9	6.4 ± 0.8	6.7 ± 0.9
	Geometric mean	6.6	6.3	6.7
	Range (median)	4.7–8.0 (6.9)	5.4–8.0 (6.0)	5.5–8.0 (6.6)
SOM (g/kg)	Mean ± SD	18.5 ± 5.8	21.9 ± 6.5	23 ± 6
	Geometric mean	17.3	21	22.2
	Range (median)	2.8–31.4 (19.3)	12.1–38.3 (21.8)	11.5–34.0 (22.8)
TN (mg/kg)	Mean ± SD	756 ± 249	973 ± 298	1034 ± 268
	Geometric mean	715	927	1000
	Range (median)	269–1298 (748)	447–1483 (984)	637–1544 (1040)
TP (mg/kg)	Mean ± SD	643 ± 196	429 ± 104	602 ± 254
	Geometric mean	616	418	561
	Range (median)	345–1098 (581)	285–757 (408)	357–1326 (487)
AP (mg/kg)	Mean ± SD	21 ± 13.9	14 ± 12.7	16.1 ± 26.7
	Geometric mean	17.6	9.9	9.2
	Range (median)	6.6–65.8 (16.5)	1.6–58.9 (11.6)	2.9–123 (6.9)
AK (mg/kg)	Mean ± SD	93.5 ± 51.4	86.3 ± 21.1	83.3 ± 51.1
	Geometric mean	84.3	84.0	74.0
	Range (median)	43.5–314 (78.9)	58.8–134 (78.9)	32.4–275 (76.5)

*R* rape field soil, *W* wheat field soil, *P* paddy field soil, *SOM* soil organic matter, *TN* total nitrogen, *TP* total phosphorus, *AP* available phosphorus, *AK* available K.

### Total concentrations and spatial distribution characteristics of metals in soils

**Figure 2:**
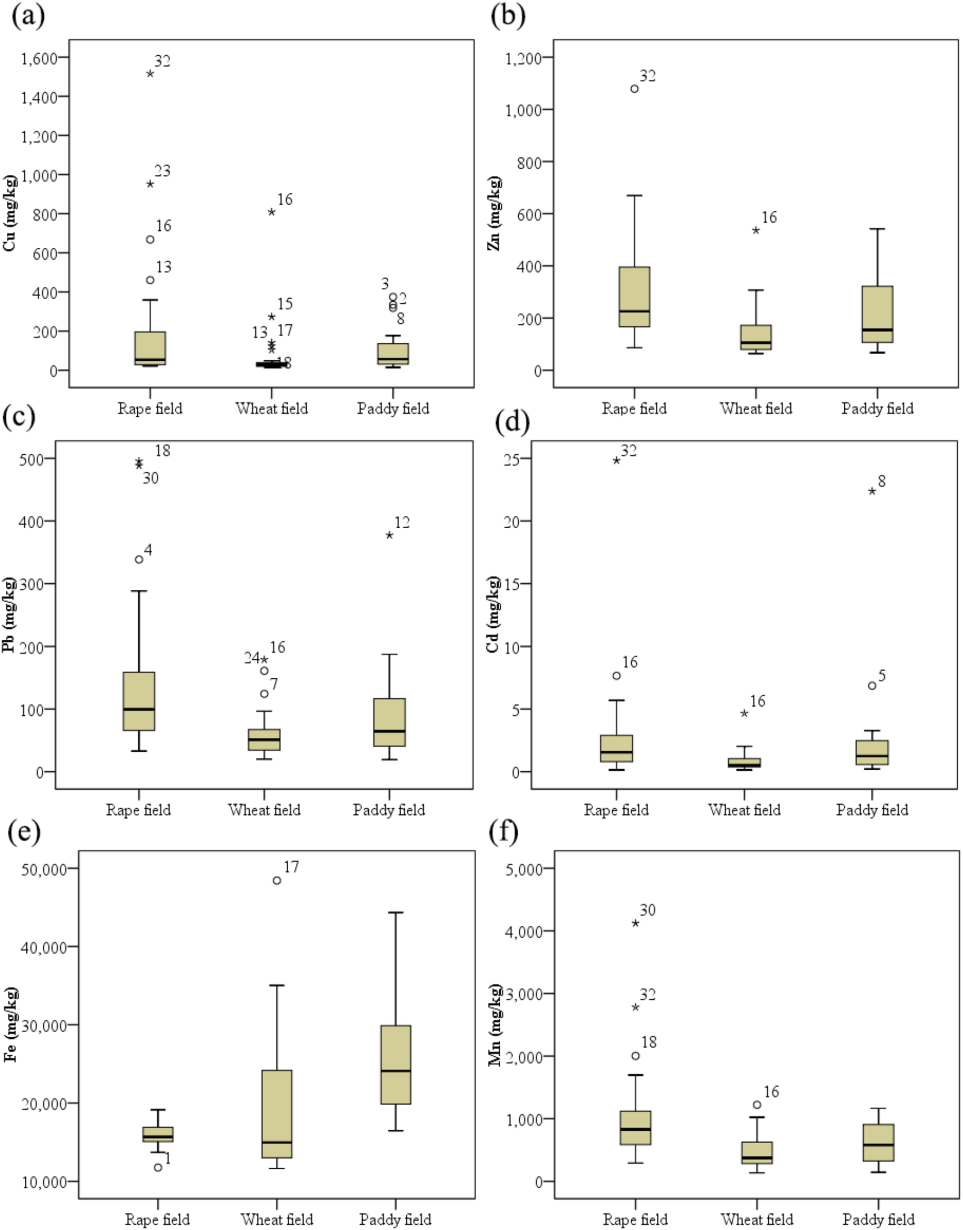
Descriptive statistics and distribution of heavy metals ((a) Cu, (b) Zn, (c) Pb, (d) Cd, (e) Fe, and (f) Mn) in soil samples collected in the study area. (Rape fields (n = 36); Wheat fields (n = 25); Paddy fields (n = 20), on dry weight basis).

Figure 2 illustrates the total concentrations of the tested metals in agricultural soil samples under different farmland use patterns. Total concentrations of metals in the 81 soil samples exhibited broad variation. Among the metals, Fe had the highest concentration, and Cu, Mn, and Zn had higher concentrations than Cd and Pb. In addition, there were differences in metal concentrations among the different farmland use patterns (Fig. 2). Generally, total Cu, Zn, Pb, Cd, and Mn concentrations in soils collected from the wheat fields were lower than those from the other two fields, and the differences in total Zn, Mn, and Pb concentrations between the rape and wheat fields were significant (*p* < 0.05).

According to GB 15618-2018^31^, the risk screening values (RSVs) of Cu, Zn, Pb, and Cd are 50, 200, 70, and 0.3 mg/kg, respectively, for pH ≤ 5.5; 50, 200, 90, and 0.3 mg/kg, respectively, for 5.5 < PH ≤ 6.5; 100, 250, 120, and 0.3 mg/kg, respectively, for 6.5 < PH ≤ 7.5; and 100, 300, 170, and 0.6 mg/kg, respectively, for pH > 7.5. In the paddy fields, the RSVs of Pb and Cd are 80 and 0.3 mg/kg, respectively, for pH ≤ 5.5; 100 and 0.4 mg/kg, respectively, for 5.5 < PH ≤ 6.5; 140 and 0.6 mg/kg, respectively, for 6.5 < PH ≤ 7.5; and 240 and 0.8 mg/kg, respectively, for pH > 7.5. Therefore, in the rape fields, 44%, 50%, 39%, and 92% of the soil samples exceeded RSVs of Cu, Zn, Pb, and Cd, respectively. In the wheat fields, 20%, 16%, 12%, and 88% of the soil samples exceeded RSVs of Cu, Zn, Pb, and Cd, respectively. In the paddy fields, 45%, 30%, 15%, and 75% of the soil samples exceeded RSVs of Cu, Zn, Pb, and Cd, respectively. Furthermore, 67%, 32%, and 55% of soil samples in the rape, wheat, and paddy fields exceeded 1 mg Cd kg^−1^ soil, and the highest value was 24.8 mg/kg. According to the results, mining operations in the region have led to soil Cd contamination under the three farmland use patterns. In addition, the rape fields had Cu, Zn, and Pb contamination, and paddy fields had Cu and Zn contamination. The results are similar to those reported in previous studies on heavy metal contamination trends in soils in the Tongling area^8,32^. However, notably, the Zn, Pb, and Cd concentration ranges in soils from the three studied fields were almost all above the local geochemical background levels; similarly, the Cu and Mn concentration ranges in most of the soil samples were also above the local geochemical background levels^26^. The average concentrations of Cu, Zn, Pb, Cd, and Mn were 2.86, 2.77, 3.43, 10.54, and 1.12-fold higher than the local geochemical background values, respectively.

**Figure 3:**
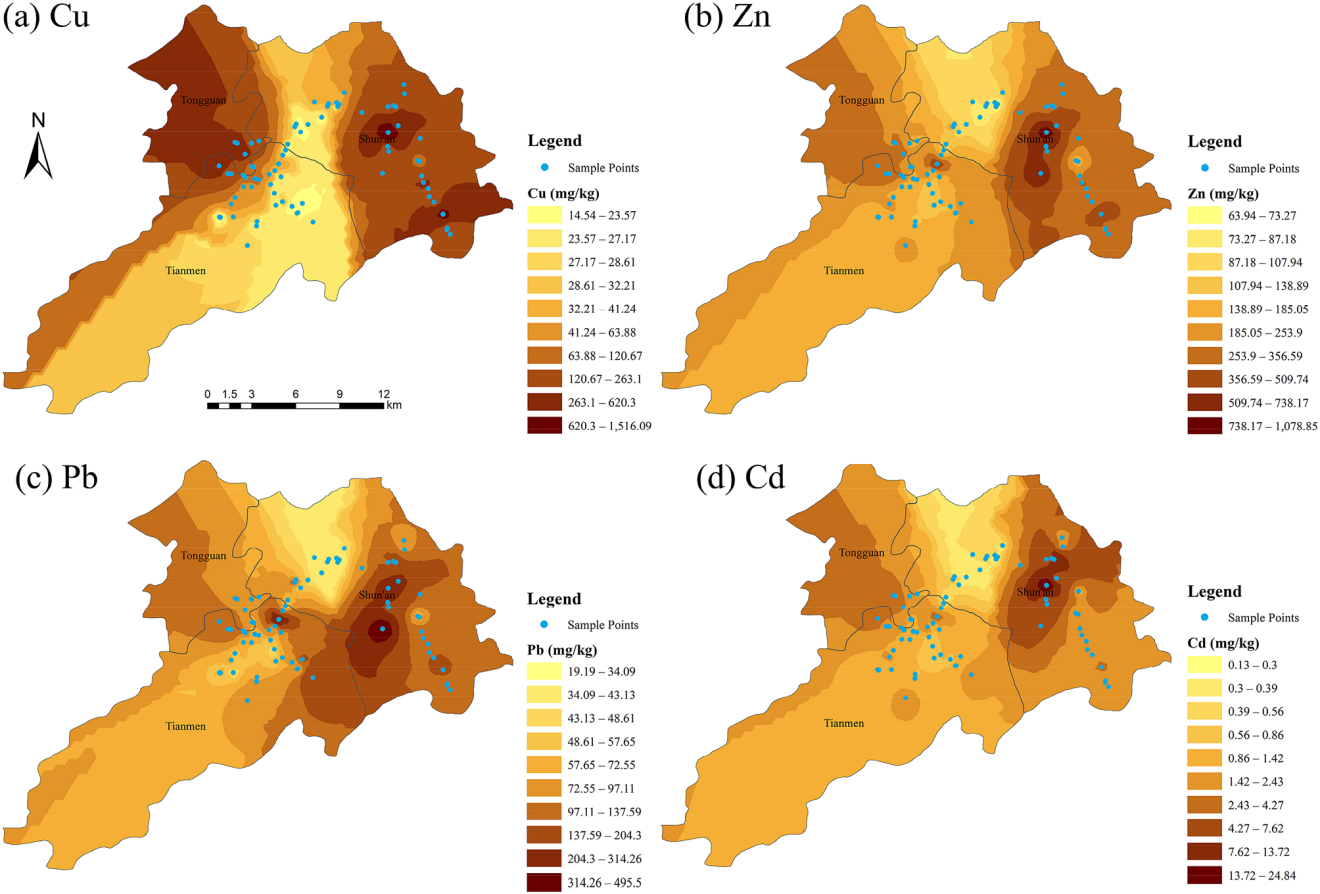
Spatial distribution maps of (**a**) Cu, (**b**) Zn, (**c**) Pb, and (**d**) Cd in the study area. The base map was generated in ArcGIS10.2 (Esri, Redlands, CA, USA, 2014) (https://www.esri.com/en-us/arcgis/about-arcgis/overview).

In order to research spatial distribution characteristics of metals in soils, spatial distribution maps of Cu, Zn, Pb, and Cd were generated using the ordinary kriging interpolation method (Fig. 3). The spatial patterns of the four metals were similar, with high concentrations mainly focused around Shunan and Tongguanshan, where the Xinqiao mine, Fenghuangshan mine, Linchong, Xiangsigu, and Yangshanchong tailing pond are found. The sites with Cu concentrations exceeding 120 mg/kg were primarily located near the above-mentioned mine sites (Fig. 3a). The sites with Zn (> 350 mg/kg), Pb (> 140 mg/kg), and Cd (> 4.0 mg/kg) were also primarily located near the above mine sites (Fig. 3b,c,d). Metal concentrations increased obviously with a decrease in distance from the above mine sites, consistent with the reports of a previous study in the Tongling mine^32^. For example, soil samples from the rape field at site 32 (R32) had extremely higher Cu, Zn, and Cd concentrations than those from other sites, which could be due to site R32 being the nearest to the Xinqiao mine (Fig. 1). Similarly, soil samples from the paddy field at site 8(P8) had extremely higher Cd concentrations than those of the other sites, which could be due to long-term irrigation using water from a river in the vicinity (Fig. 1).

### Identification of sources of soil heavy metals based on the principal component analysis

Loadings of heavy metals and soil physicochemical properties based on principal component analysis (PCA) for the 81 soil samples are shown in Table 2. When the PCA was performed based only on the data of six heavy metal concentrations, two principal components (PCs) were found (eigenvalues > 1). These two components could explain 76.67% of the total variance. The first PC (PC1) had high factor loadings for Cu, Zn, Pb, Cd, and Mn, which could explain 59.80% of the total variance. Correlation analysis also showed that Cu, Zn, Pb, Cd, and Mn were highly correlated with each other (data table not presented), indicating that they originated from similar sources. The second PC (PC2) explained 16.87% of the total variance and characterized by a positive Fe loading. In addition, Fe exhibited poor correlations with Cu, Zn, Pb, Cd, and Mn. In the present study, Fe had the highest concentration in all soil samples, considering Fe is the fourth most abundant element in the Earth’s crust; nevertheless, the Fe contents were similar to soil Fe contents in other parts of southern China^33^. Therefore, Fe concentrations were mainly influenced by natural factors, and PC2 could largely be attributed to non-anthropogenic sources. In addition, mean Cu, Zn, Pb, Cd, and Mn concentrations were all above the local geochemical background levels, so that PC1 may be better attributed to anthropogenic sources, since mining activities are undertaken in the study area.

**Table 2:** Loadings of heavy metals and soil physicochemical properties on principal components for 81 soil samples.

	Principal components			Principal components			
	1	2		1	2	3	4
Cu	0.778	0.238	Cu	0.754	0.130	− 0.226	− 0.102
Zn	0.968	− 0.029	Zn	0.936	− 0.011	− 0.238	− 0.030
Pb	0.789	− 0.342	Pb	0.731	− 0.169	− 0.271	0.121
Cd	0.820	0.118	Cd	0.751	0.263	− 0.344	0.069
Fe	0.289	0.861	Fe	0.353	0.413	0.301	− 0.623
Mn	0.817	− 0.285	Mn	0.805	− 0.286	− 0.146	0.084
Eigenvalue	3.588	1.012	pH	0.593	0.183	0.364	− 0.325
Variance contributes/%	59.799	16.867	SOM	0.361	0.782	0.274	0.199
Contribution of accumulated variance/%	59.799	76.666	TN	− 0.152	0.781	0.122	0.406
			TP	0.683	− 0.328	0.482	0.007
			AP	0.292	− 0.444	0.707	0.274
			AK	0.384	0.013	0.078	0.560
			Eigenvalue	4.522	1.928	1.372	1.126
			Variance contributes/%	37.683	16.066	11.430	9.380
			Contribution of accumulated variance/%	37.683	53.749	65.179	74.558

We also conducted a PCA based on the heavy metals and soil physicochemical property data (Table 2). According to the results, the eigenvalues of the first four PCs were all > 1, which could explain 74.56% of the total variance. PC1 had strong positive Cu, Zn, Pb, Cd, Mn, pH, and TP loadings, explaining 37.68% of the total variance. SOM and TN were associated with PC2, indicating that PC2 reflected natural factors related to soil occurrence^34^. PC3 was strongly correlated with AP, whereas PC4 was strongly correlated with AK, indicating that PC3 and PC4 mainly reflected the impacts of land use^34^. Considering Cu, Zn, Pb, Cd, and Mn were attributed to PC1, and the metals exhibited poor correlations with natural and land use factors, the concentrations of the metals were considered to be mainly influenced by mining activities.

### Extractable metal concentrations in soils

The concentrations of the metals extracted using five methods are summarized in Table 3. The concentrations of extractable metals were influenced significantly by the farmland use patterns and the types of extractants used. In the rape fields, concentrations of all the metals extracted using EDTA were higher than those extracted with the other four types of extractants. Similarly, in the wheat and paddy fields, Cu, Pb, and Mn concentrations extracted using EDTA were higher than those extracted with the other four types of extractants, whereas Zn, Cd, and Fe concentrations extracted using 0.1 mol/L HCl were higher than those extracted using the other extractants. Following extraction with DTPA and EDTA, the concentrations of the metals in most soil samples collected from the rape fields were in the order of Mn > Fe > Pb > Cu > Zn > Cd, and the concentrations of metals in most soil samples collected from the wheat and paddy fields were in the order of Fe > Mn > Pb > Cu > Zn > Cd. However, independent of the farmland use patterns, the concentrations of all the metals extracted using NH_4_OAC and NH_4_NO_3_ were much lower than those extracted with the other three types of extractants.

**Table 3:** Extractable metal concentrations in the soils (mg/kg).

Soil type	Extraction methods	Cu	Zn	Pb	Cd	Fe	Mn
		Range (median)	Range (median)	Range (median)	Range (median)	Range (median)	Range (median)
R	DTPA (n = 36)	1.3–253 (8.2)	1.3–32.9 (5.3)	2.2–92.0 (11)	0.05–5.2 (0.27)	18.6–166 (43)	8.5–191 (46)
	EDTA (n = 36)	1.9–409 (15.0)	1.9–149 (8.7)	5.1–192 (27.4)	0.06–8.0 (0.4)	55.3–480 (162)	53.6–2056 (207)
	HCl (n = 33)	1.7–150 (7.1)	2.3–41.5 (9.7)	2.4–79.7 (10.5)	0.07–2.7 (0.3)	22.4–534 (118)	41.0–276 (92)
	NH_**4**_**OAC** (n = 36)	Ud-31 (0.42)	Ud-5.9 (0.14)	Ud-4.5 (0.07)	0.03–1.5 (0.11)	Ud-1.8 (0.03)	0.08–104 (20.6)
	NH_**4**_**NO**_**3**_ (n = 36)	Ud-14.9 (0.04)	Ud-15.0 (0.7)	Ud-8.8 –	Ud-0.5 (0.02)	Ud-4.7 –	0.08–133 (20.9)
W	DTPA (n = 25)	1.7–145 (5.0)	1.2–24.7 (3.3)	3.2–47.8 (7.8)	0.06–1.1 (0.2)	16.4–90.8 (43.3)	7.5–79 (23.2)
	EDTA (n = 25)	3.6–321 (8.4)	1.4–64.9 (3.4)	5.3–93.0 (15.5)	0.05–1.6 (0.19)	68.6–354 (152)	16.5–690 (100)
	HCl (n = 24)	2.4–231 (6.3)	2.3–48.2 (4.9)	3.7–47.5 (9.8)	0.08–1.6 (0.22)	67.2–369 (174)	20.8–181 (72.3)
	NH_**4**_**OAC** (n = 25)	0.1–10.4 (0.26)	Ud-1.7 (0.18)	Ud-2.4 –	0.02–0.6 (0.06)	Ud –	0.8–41.6 (7.5)
	NH_**4**_**NO**_**3**_ (n = 25)	Ud-1.5 (0.04)	0.04–4.4 (1.2)	Ud-2.7 (0.1)	Ud-0.2 (0.04)	Ud-1.8 (0.23)	0.3–54.8 (11.5)
P	DTPA (n = 20)	1.8–123 (13.7)	0.8–31.3 (5.3)	3.8–105 (12.2)	0.07–11 (0.33)	24.6–324 (112)	5.5–69.6 (15.5)
	EDTA (n = 20)	5.0–223 (24.5)	1.6–74.2 (7.8)	8.5–177 (25.2)	0.07–15 (0.43)	52.2–503 (255)	17.7–794 (179)
	HCl (n = 18)	3.6–173 (14.0)	2.7–53.2 (11.3)	3.1–95.8 (13.6)	0.1–13.4 (0.4)	16.1–1060 (335)	14.5–383 (104)
	NH_**4**_**OAC** (n = 20)	Ud-6.3 (0.3)	Ud-10.2 –	Ud-3.3 (0.2)	0.01–3.7 (0.1)	Ud –	0.02–15.3 (3.7)
	NH_**4**_**NO**_**3**_ (n = 20)	Ud-2.2 (0.08)	Ud-30.4 (0.5)	Ud-2.4 (0.02)	Ud-1.4 (0.05)	Ud-0.2 –	0.2–35.5 (4.7)

*Ud* under detection limit, *–* more than half of samples did not exceed the detection limit.

The concentrations of 0.1 mol/L HCl-extracted heavy metals in soils from the rape fields at the R21, R23, and R32 sites, in the wheat fields at the W21 site, and in the paddy fields at the P1 and P2 sites were extremely low, especially in the cases of Fe and Pb. For example, 0.1 mol/L HCl-extracted Fe concentrations at the R21, R23, and R32 sites were 0.2 mg/kg, 0.6 mg/kg, and below the detection limit, respectively, whereas the range of 0.1 mol/L HCl-extracted Fe concentration in the other sites in the rape fields was 22.4–533 mg/kg. Therefore, 0.1 mol/L HCl-extracted heavy metal concentrations at the R21, R23, R32, W21, P1, and P2 sites were omitted from subsequent analyses to ensure homogeneity of variance. The basic pH values at the sites (pH values at R21, R23, R32, W21, P1, and P2 were 8.0, 8.0, 7.7, 8.0, 8.0, and 8.0, respectively) could explain this low HCl-extractability and reduced mobility^35,36^. Although HCl is considered a universal extractant, it may not be suitable under alkaline soil conditions.

### Heavy metal concentrations in rape, wheat and rice grains

**Figure 4:**
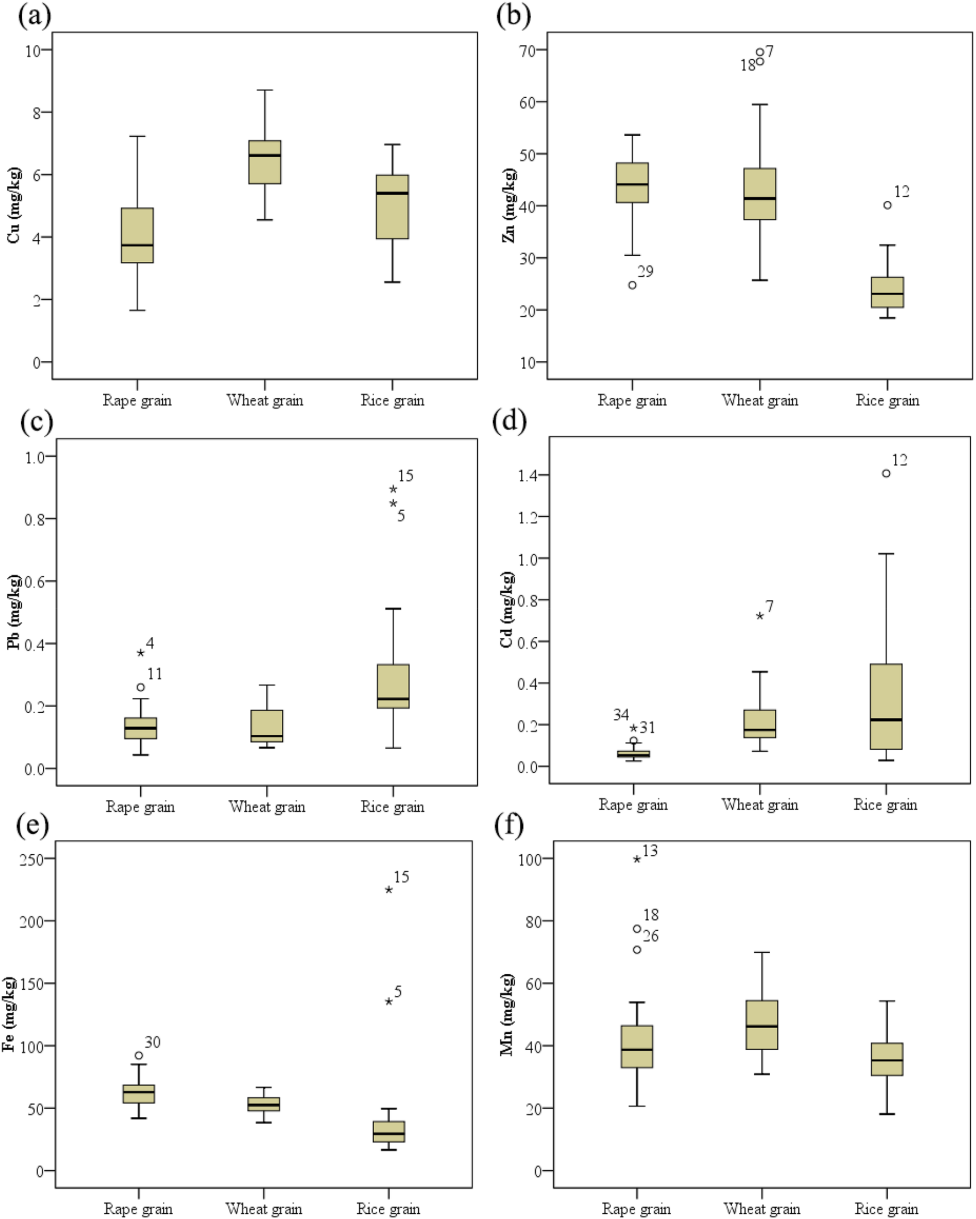
Concentrations of heavy metals ((a) Cu, (b) Zn, (c)Pb, (d) Cd, (e) Fe, and (f) Mn) in the grain of three different crops. (Rape grains (n = 36); Wheat grains (n = 25); Rice grains (n = 20), on dry weight basis).

Figure 4 illustrates the concentrations of six heavy metals in rape, wheat, and rice grains. Similar to the order of the soil metal concentrations, the Fe, Mn, Zn, and Cu concentrations in the grains were much higher than the Pb and Cd concentrations.

The results are similar to those reported in previous studies with regard to heavy metal concentration trends in rice grains^16,37^. The reason could be Fe, Mn, Zn, and Cu are all essential for crop growth as micronutrients, leading to the higher levels in soils. Among the studied crops, rice grains accumulated relatively lower amounts of Fe, Mn, and Zn than rape and wheat grains, where Pb and Cd concentrations exhibited opposite trends. The trends are consistent with the findings of Liu et al.^38^ and Du et al.^1^, and indicate that rice has a stronger Cd uptake capacity from soil. Williams et al.^39^ also reported higher rice Cd concentrations than wheat, barley, and maize Cd concentrations. The results could also be attributed to water management and soil oxidation–reduction status in soil-rice systems. Paddy fields are irrigated considerably more than wheat and rape fields. In addition, the water in the study area was contaminated by Cd, Cu, As, and Zn^27^. Previous studies have also reported that water management practices influence Cd uptake by rice and its bioavailability in soils^19,20,40^. In the cases of Zn and Pb, according to Feng et al.^23^, rice grains accumulated lower amounts of Zn than wheat grains, whereas rice grains accumulated higher Pb amounts than wheat grains. Although soil Cd concentrations were generally high in the rape fields, the concentrations of Cd in rape grains were lower than those in the wheat and rice grains.

On the other hand, Cu concentrations in grains in all the three crops were below the maximum allowable Cu levels in food (10 mg/kg (GB 15199-94)). Similarly, Zn concentrations in rice grains were below the maximum allowable Zn levels in food (50 mg/kg (GB 13106-91)), while 25% of the rape grain samples and 20% of the wheat grain samples exceeded the threshold value. Although total Pb concentrations in soils were generally low in the three farmland use patterns, Pb concentrations in 70% of the rice grain samples exceeded the maximum allowable Pb levels in food (0.2 mg/kg (GB 2762-2005)). Only four rape and two wheat grain samples exceeded the Pb threshold values, respectively. The varying Pb trends are potentially linked to physical contamination from direct atmospheric deposition^8^, differences in physiological activities among the crops, and the fruit structures of the studied crops^23^. Similar to the soil Cd contamination, 96% and 60% of wheat and rice grain samples, respectively exceeded the maximum allowable Cd levels in food (0.1 mg/kg and 0.2 mg/kg for wheat and rice, respectively (GB 2762-2005)). Furthermore, Cd concentrations in approximately 10% of the rice grain samples exceeded 1.0 mg/kg. Conversely, only 14% of rape grain samples exceeded the maximum allowable Cd levels in food (Cd: 0.1 mg/kg for rape (GB 2762-2005)), and the maximum Cd concentration in rape grains was 0.18 mg/kg. According to the results, rape grains were generally safe for consumption whereas wheat and rice grains posed health threats in the study area.

### Soil to grain bioaccumulation factors

**Figure 5:**
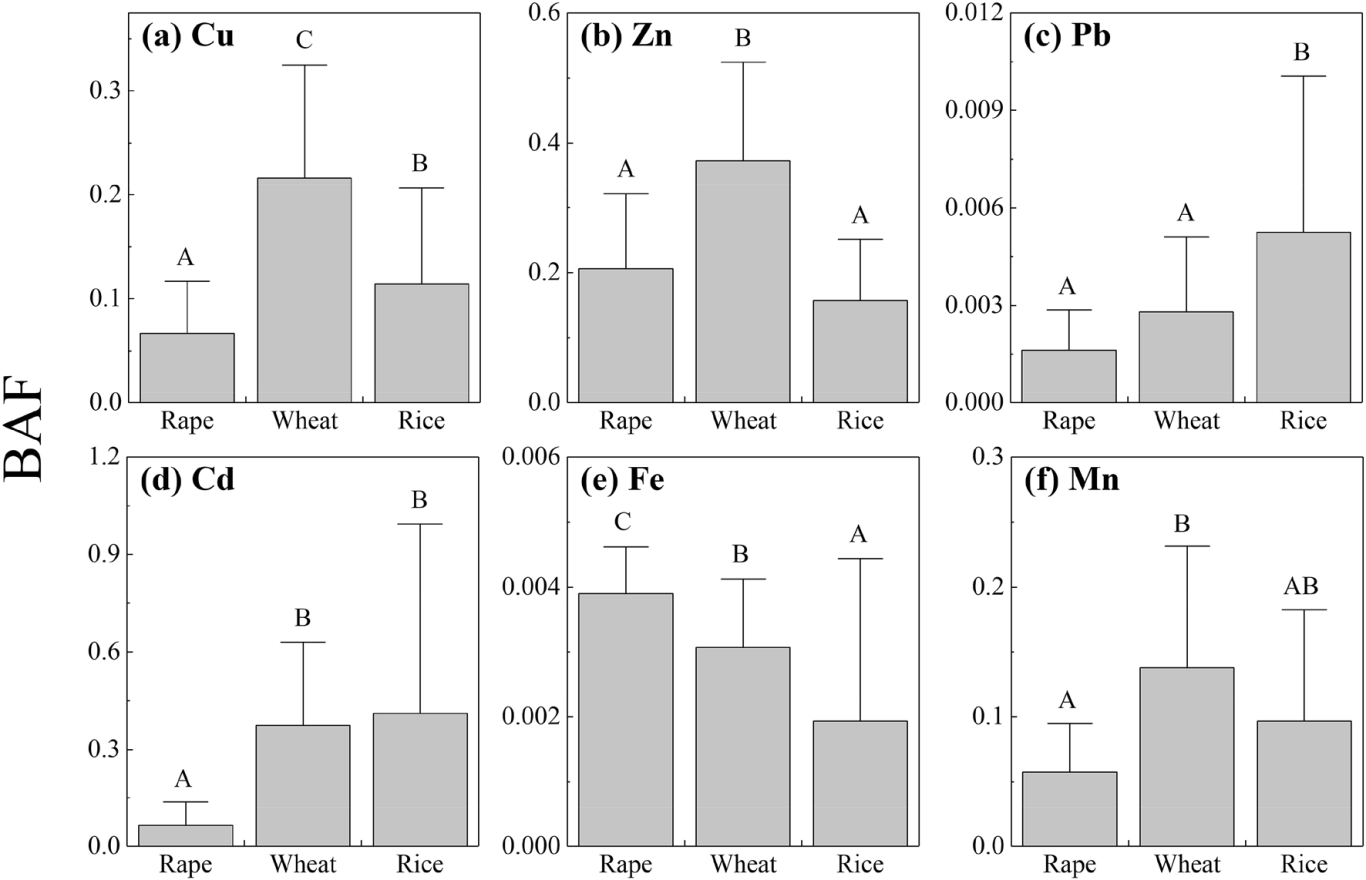
Bioaccumulation factors (*BAF*) of heavy metals ((a) Cu, (b) Zn, (c) Pb, (d) Cd, (e) Fe, and (f) Mn) from soil to the grains of three crop species. The error bars indicate the standard deviation. Different letters on bars indicate significant difference (*p* < 0.05) between means.

*BAF* values have been used widely to evaluate the capacity of crop grains to accumulate metals from soil^30,41^. Similar to a previous study on food crops^37^, Fe and Pb had the lowest *BAF* values (Fig. 5). Generally, metal accumulation in crop grains did not increase considerably with an increase in total concentrations of metals in soil. Heavy metal accumulation could have been regulated by crops, so that only low amounts were accumulated into grains. In addition, there were significant differences in some *BAF* values of the same metal across different crop species (Fig. 5). Different crop species have different accumulation capacities for the same metal^42^. Overall, the average *BAF* values of Cu (0.22), Zn (0.37), and Mn (0.14) in wheat grains were significantly higher than those in rape and rice grains. The average *BAF* value of Pb (0.005) in rice grains was significantly higher than those in rape and wheat grains, and the average *BAF* values of Cu (0.07) and Cd (0.06) in rape grains were significantly lower than those in wheat and rice grains. The results indicated that rape grains have lower heavy metal accumulation capacity than wheat and rice grains, except in the cases of Zn and Fe. However, the finding is not consistent with the results of a previous study^43^, which report that grasses have lower accumulation capacity than dicotyledonous plants. Nevertheless, as mentioned above, numerous factors could influence the accumulation capacity of metals in crop grains.

## Discussion

### Geochemistry and source characteristics of soil heavy metals in the study area

Compared to the risk screening value of soil pollution in agricultural land (GB 15618-2018)^31^ and the local geochemical background value^26^, the results of the present study indicated that the three fields were contaminated to some degree, especially by Cd. The average concentrations of Cu, Zn, Pb, and Cd in the studied soils were significantly higher than those reported in soils from the nearest area in China, including Anhui Chaohu^44^, Anhui Wuhu^45^, and Jiangsu^41^. For example, Qin et al.^44^ reported that the average concentrations of Cu, Zn, Pb, and Cd in soils in the north of Chaohu were 24.47, 57.25, 28, and 0.13 mg/kg, respectively. The maximum concentrations of Cu, Zn, Pb, and Cd in the studied soils were also higher than those reported by Wang et al.^46^ around a tungsten–molybdenum mine, although the average concentrations were comparable to or lower than those reported in the tungsten–molybdenum mine. Furthermore, in soils from paddy fields, the average concentrations of Cd reported by Hao et al.^47^ on a copper mine area and Cui and Du^48^ in an abandoned lead–zinc mine were both lower than those observed in the present study. The results suggest that the soil in the study area is under the influence of exogenous heavy metal pollution.

The coefficients of variation (CV) of the studied heavy metals were all > 50%, except for Fe. Higher CVs indicate that the spatial distribution of metals is uneven due to the influence of anthropogenic factors^49,50^. Spatial distribution maps (Fig. 3) showed that high concentrations of metals were mainly observed near Xinqiao mine, Fenghuangshan mine, Linchong, Xiangsigu, and Yangshanchong tailings pond (Fig. 1). Metal concentrations increased with a decrease in distance from the above mine sites, indicating that the mine sites are the major sources of heavy metals in the study area. Previous studies have also reported similar spatial distribution trends for soil metals around other metal mines^51,52^. Windborne transport and water-runoff erosion of the mine tailings are key pathways for metals to the surrounding soils^50,52^. Yun et al.^50^ and Li et al.^51^ investigated the distribution and transport of metals in other metal mines, and revealed that transport and distribution of metals were controlled primarily by wind and water-runoff erosion processes of mine tailings. Therefore, Cu, Zn, Pb, and Cd pathways to the surrounding soils in the present study could be attributed to wind and water-runoff erosion of the mine tailings, since mining activities take place in the study area.

The results of PCA (Table 2) showed that PC1 had high factor loadings for Cu, Zn, Pb, Cd, and Mn, while Fe was associated with PC2. In addition, Cu, Zn, Pb, Cd, and Mn were highly correlated, whereas Fe exhibited poor correlations with the other metals. The results suggested that Fe and Cu, Zn, Pb, Cd, and Mn had different sources, and Cu, Zn, Pb, Cd, and Mn were likely attributable to the same sources. As mentioned above, Fe was associated with a relatively low CV, indicating that Fe was potentially influenced by natural sources^53^. Therefore, Cu, Zn, Pb, Cd, and Mn were mainly influenced by anthropogenic sources, which is consistent with the larger CV values observed for the metals. Similar results on the sources of Cu, Zn, Pb, and Cd in soils around the mine area have been reported by other researchers^51,53^. On the other hand, some previous studies^53,54^ have demonstrated that fertilizer supplementation is associated with high metal concentrations in soil, but mining activities are the predominant sources. In the present study, according to the PCA results (Table 2), Cu, Zn, Pb, Cd, and Mn exhibited poor correlations with natural and land use factors, and mining activities were their major sources. Rodriguez et al.^55^ also demonstrated that high concentrations of metals around the San Quintin Pb–Zn mine were not associated with land use, and concluded that mining activities were their main sources.

### Relationships between soil properties and metal extractability

Soil properties could influence metal bioavailability through their influence on metal extractability. In addition, soil properties influence soil extractable metal concentrations differently in different studies^14,16^. Table S1, S2, and S3 show the correlation coefficients between soil properties and extractable metal concentrations and AI of metals in soil. The relationships were different across different farmland use patterns, extraction methods, metals, and the soils with different properties. For example, AK had poor correlations with all extractable metal concentrations and soil metal AIs in rape and paddy fields, while it exhibited significant correlations with most extractable metal concentrations or soil metal AIs in the wheat fields. The divergence could be due to different fertilizer management practices under the different farmlands. In the three studied fields, soil NH_4_OAC and NH_4_NO_3_-extractable metal concentrations exhibited poor correlations with SOM, while some DTPA, EDTA and HCl-extractable metal concentrations showed significant correlations with SOM. The variation could be due to differences in the chemical properties of neutral salt extractants and complexing agents. According to Fang et al.^56^ and Wang et al.^57^, SOM was the key factor influencing DTPA or EDTA-extractable metal concentrations. Independent of the farmland use patterns, the key soil parameter that influenced metal extractability was soil pH, especially following NH_4_OAC and NH_4_NO_3_ extraction. In addition, soil pH had a significant negative correlation with NH_4_OAC and NH_4_NO_3_-extractable Zn, Pb, Cd, and Mn concentrations and the AIs of the metals. DTPA-extractable Fe concentration and its AI also had significant negative correlations with soil pH.

In the present study, total heavy metal concentrations often influenced extractable concentrations of heavy metals in soils. Table 4 shows the interactive effects of soil properties, including total metal concentration, on extractable heavy metal concentrations in soils. For simplicity, only r^2^ values and the entered variables are listed in Table 4. In the three studied fields, the total metal concentrations and soil properties were all included in the assessment models for soil DTPA, EDTA, and HCl-extractable Cu, Zn, Pb, and Cd concentrations as the first and second variables, respectively. In the cases of NH_4_OAC and NH_4_NO_3_-extractable Cu, Zn, Pb, and Cd, only soil total metal concentrations or combinations of soil total metal concentrations and soil pH were included in the multiple regression equations, excluding soil NH_4_OAC-extractable Zn in the paddy fields (TP instead of pH). Notably, the combination of soil properties and total metal concentrations did not improve the regression coefficients of assessment models of soil DTPA, EDTA, and HCl-extractable metal concentrations significantly. However, the soil pH and total metal combination improved the regression coefficients for the estimation of NH_4_OAC and NH_4_NO_3_-extractable Zn, Pb, and Cd concentrations significantly, especially in the case of NH_4_NO_3_ methods, where soil pH was the first variable in the multiple regression equations (Table 4). Therefore, independent of the farmland use patterns, soil DTPA, EDTA, and HCl-extractable Cu, Zn, Pb, and Cd concentrations were controlled mainly by soil total metals, while NH_4_OAC and NH_4_NO_3_-extractable Zn, Pb, and Cd concentrations were controlled mainly by soil pH or a combination of soil pH and total metals. Similar results on the factors influencing soil DTPA-extractable metal concentrations have been supported by other researchers^14,15^. It may be associated with the fact that some metals associated with the soil components could be extracted by the complexing agents, even though the solubility of some metals was low. The results of the influence of soil pH on soil NH_4_OAC and NH_4_NO_3_-extractable metal concentrations are similar to those of other studies^8,58^. The significant negative correlations between soil pH and NH_4_OAC and NH_4_NO_3_-extractable Zn, Pb, Cd, and Mn concentrations, in addition to the AIs of the metals, could be linked to differences in hydrolysis, displacement, and precipitation of metal ions in high or low pH soil. Soil pH had minimal effect on NH_4_OAC and NH_4_NO_3_-extractable Cu concentrations. The weak relationships between neutral salt extractable-Cu and soil pH have also been reported in previous studies^8,56^. The results could be attributed to the strong affinity for SOM by Cu. For soil DTPA-extractable Fe concentrations, only soil pH was included in their multiple regression equations in the three fields; furthermore, total Fe was not included in any multiple regression equation based on the other extractable methods. The results suggest that soil DTPA-extractable Fe concentrations are controlled mainly by soil pH.

**Table 4:** Stepwise multiple regressions between soil extractable metals and soil properties (including soil total metals).

Extractant		Regres. steps	Cu	r^2^	Zn	r^2^	Pb	r^2^	Cd	r^2^	Fe	r^2^	Mn	r^2^
DTPA	R	1	TCu	0.916***	TZn	0.664***	TPb	0.850***	TCd	0.761***	− pH	0.500***	− pH	0.313**
		2	− pH	0.930***	Ap	0.708***	TP	0.911***					TMn	0.473***
	W	1	TCu	0.950***	TZn	0.726***	TPb	0.836***	TCd	0.801***	− pH	0.642***	ns	
		2	SOM	0.977***			SOM	0.896***	− pH	0.848***				
	P	1	TCu	0.884***	TZn	0.680***	TPb	0.826***	TCd	0.929***	− pH	0.591***	ns	
		2	− TP	0.941***	− pH	0.748***	− pH	0.873***	− TP	0.970***				
EDTA	R	1	TCu	0.921***	TZn	0.712***	TPb	0.887***	TCd	0.826***	TN	0.239**	TMn	0.540***
		2	TN	0.948***	TN	0.763***	TP	0.911***					TP	0.675***
	W	1	TCu	0.952***	TZn	0.803***	TPb	0.810***	TCd	0.848***	ns		TMn	0.910***
		2	SOM	0.980***			− pH	0.893***						
	P	1	TCu	0.925***	TZn	0.781***	TPb	0.928***	TCd	0.953***	ns		TMn	0.644***
		2	TN	0.956***	TN	0.841***	AK	0.953***	TP	0.975***			TP	0.754***
HCl	R	1	TCu	0.941***	TZn	0.746***	TPb	0.663***	TCd	0.801***	TFe	0.145*	TMn	0.439**
		2			Ap	0.789	Ap	0.769					TN	0.520**
	W	1	TCu	0.960***	TZn	0.846***	TPb	0.825***	TCd	0.863***	− pH	0.289**	TMn	0.760***
		2	SOM	0.979***			SOM	0.872***					− pH	0.816***
	P	1	TCu	0.853***	TZn	0.837***	TPb	0.584***	TCd	0.962***	Ap	0.453***	TMn	0.744***
		2	TP	0.947***	TN	0.890***	TP	0.860***	TP	0.985***			TP	0.819***
NH_**4**_**OAC**	R	1	TCu	0.665***	TZn	0.166*	TPb	0.270**	TCd	0.392***			− pH	0.414***
		2			− pH	0.403***	− pH	0.631***	− pH	0.577***			− Ap	0.539***
	W	1	TCu	0.879***	ns		TPb	0.281**	TCd	0.235**			− pH	0.551***
		2					− pH	0.611***	− pH	0.554***			TMn	0.755***
	P	1	TCu	0.655***	TZn	0.250*	− pH	0.454**	TCd	0.681***			− Ap	0.488**
		2	− pH	0.785***	− TP	0.538**	TPb	0.802***	− pH	0.920***				
NH_**4**_**NO**_**3**_	R	1	TCu	0.190*	− pH	0.557***	− pH	0.573***	− pH	0.396**			− pH	0.498***
		2	− pH	0.370**	TZn	0.739***	TPb	0.64***	TCd	0.680***			− Ap	0.602***
	W	1	TCu	0.413***	− pH	0.734***	− pH	0.748***	− pH	0.644***			− pH	0.714***
		2			TZn	0.894***	TPb	0.848***	TCd	0.865***			TMn	0.855***
	P	1	TCu	0.428**	− pH	0.790***	− pH	0.842***	− pH	0.377**			− pH	0.636***
		2			TZn	0.879***	TPb	0.916***	TCd	0.77***			TMn	0.754***

*TCu* total Cu, *TZn* total Zn, *TPb* total Pb, *TCd* total Cd, *TFe* total Fe, *TMn* total Mn.

***, ** and * indicate significant correlations at the probability level of *p* < 0.001, *p* < 0.01 and *p* < 0.05, respectively, *ns* not significant.

Lopes et al.^14^ also reported that extractable Fe concentrations are mainly influenced by soil pH but not total Fe concentrations in soils. Other studies have also reported that high soil pH could reduce Fe bioavailability^59,60^, and the trends could be driven by the conversion of different forms of Fe under high or low soil pH. Notably, the models of metals extracted with HCl had r^2^ values lower than those associated with other extraction techniques (data not shown in Table 4). However, when soil samples with high pH values were deleted, r^2^ values of the models increased remarkably (Table 4). Therefore, HCl-extractable methods were not included in subsequent analyses and discussions.

### Relationships between soil properties and metal accumulation in crop grains

The relationship between metal concentrations in crop grains and soils varied based on crop species, metals, and extraction methods (Table S4, rice grains are not shown in Table S4 since no significant correlations were observed). Most of the correlation coefficients were positive, and the correlation coefficients calculated for wheat grains were always higher than those calculated for rape and rice grains (Table S4). For example, significant positive correlations were observed between Cu concentrations in wheat grains and DTPA, EDTA, NH_4_OAC, NH_4_NO_3_-extractable Cu, and total concentrations of Cu in soils. Similarly, Zn concentrations in wheat grains were significantly positively correlated with DTPA, EDTA, and NH_4_OAC-extractable Zn, and total concentrations of Zn in soils. Furthermore, there were significant positive correlations between Cd and Mn concentrations in wheat grains and in soils extracted by DTPA, NH_4_OAC and NH_4_NO_3_. Therefore, all the above extraction methods were suitable for the bioavailability analysis of the metals in soil and their bioaccumulation in wheat grains. Conversely, poor correlations were observed between Pb and Fe concentrations in the three crop species and total or extractable Pb and Fe concentrations in soils.

Stepwise multiple regression analyses were used to explore the multiple influences of soil properties on metal accumulation in crop grains (Table 5). Similar to the results of simple correlation analyses, the coefficients of soil metal variables included in the regression models were positive, indicating that increases in metal concentrations in soils would promote metal accumulation in crop grains. The results also showed that metal accumulation in wheat grains could be better explained by soil heavy metal concentrations than in rape and rice grains. That is, 38%, 25%, 30%, and 66% of the Cu, Zn, Cd, and Mn concentrations, respectively, in wheat grains, could be explained by the total Cu, NH_4_OAC-extractable Zn, DTPA-extractable Cd, and NH_4_OAC-extractable Mn concentrations in soils, respectively. Furthermore, in the second step, soil AK, total Zn, pH, and DTPA-extractable Mn were included in the Cu, Zn, Cd, and Mn accumulation models for wheat grains, respectively, and all the r^2^ values increased. The results indicated that soil pH, AK, and mixed extraction methods maybe provide more information for explaining metal accumulation in wheat grains. According to the symbol of variables, high soil pH and AK restricted Cd and Cu accumulation in wheat grains, respectively. Wang et al.^17^ observed that high soil pH restricted Cd accumulation in wheat grains, whereas Luo et al.^12^ reported that Cu uptake in wheat grains could be better explained based on mixed extraction methods.

**Table 5:** Summary of stepwise multiple regression equations for metal concentrations in the grains of three crop species as function of soil total or extractable metal concentrations and soil properties.

	Equation	*r*^*2*^	*r*_*adj*_^2^	*p*_value_
*Brassica juncea*/Rape (n = 36)	Log_10_^[grain]^ ^−^ ^(Cu)^* = 0.304 + 0.146 Log_10_^[total]^ ^−^ ^(Cu)^**	0.283	0.262	0.001
	Log_10_^[grain]^ ^−^ ^(Cd)^ = − 1.189 + 0.158Log_10_^[EDTA]^ ^−^ ^(Cd)^	0.135	0.109	0.028
	Log_10_^[grain]^ ^−^ ^(Mn)^ = 1.221 + 0.234 Log_10_^[DTPA]^ ^−^ ^(Mn)^	0.306	0.286	< 0.001
*Triticum aestivum*/Wheat (n = 25)	Log_10_^[grain]^ ^−^ ^(Cu)^ = 0.655 + 0.096Log_10_^[total]^ ^−^ ^(Cu)^	0.408	0.382	0.001
	Log_10_^[grain]^ ^−^ ^(Cu)^ = 1.192 + 0.144Log_10_^[total]^ ^−^ ^(Cu)^ − 0.318 Log_10_^(AK)^	0.560	0.520	< 0.001
	Log_10_^[grain]^ ^−^ ^(Zn)^ = 1.686 + 0.061Log_10_^[NH^_4_^OAC]^ ^−^ ^(Zn)^	0.285	0.254	0.006
	Log_10_^[grain]^ ^−^ ^(Zn)^ = 1.338 + 0.046Log_10_^[NH^_4_^OAC]^ ^−^ ^(Zn)^ + 0.559 Log_10_^[total]^ ^−^ ^(Zn)^	0.415	0.362	0.003
	Log_10_^[grain]^ ^−^ ^(Cd)^ = − 0.404 + 0.441Log_10_^[DTPA]^ ^−^ ^(Cd)^	0.330	0.301	0.003
	Log_10_^[grain]^ ^−^ ^(Cd)^ = 0.632 + 0.601Log_10_^[DTPA]^ ^−^ ^(Cd)^ − 0.145pH	0.563	0.524	< 0.001
	Log_10_^[grain]^ ^−^ ^(Mn)^ = 1.491 + 0.183Log_10_^[NH^_4_^OAC]^ ^−^ ^(Mn)^	0.674	0.659	< 0.001
	Log_10_^[grain]^ ^−^ ^(Mn)^ = 1.644 + 0.273Log_10_^[NH^_4_^OAC]^ ^−^ ^(Mn)^ − 0.167 Log_10_^[DTPA]^ ^−^ ^(Mn)^	0.732	0.708	< 0.001
*Oryza sativa*/rice (n = 20)	Log_10_^[grain]^ ^−^ ^(Cu)^ = 0.186 + 0.074 pH	0.285	0.246	0.015
	Log_10_^[grain]^ ^−^ ^(Zn)^ = 1.745–0.199 Log_10_^(AK)^	0.228	0.185	0.036

*: [grain] − (Cu) means Cu concentrations in grains; **: [total] − (Cu) means total Cu concentrations in soils.

The results of the present study also showed that Mn accumulation in wheat grains could be better explained by soil Mn concentrations when compared to the other metals. The results could be attributed to the solubility of Mn, it can form organic ligands or anionic complexes even under alkaline pH conditions^61^. Generally, not only soil heavy metal concentrations but also soil properties and combined extraction methods should be considered when estimating the bioaccumulation of some metals in wheat grains. Although rice grain metal concentrations were not significantly correlated with soil total or extractable metal concentrations, 19% Cu and 18% Zn accumulation in rice grains could be explained only by pH and AK, respectively, with high soil AK restricting Zn bioaccumulation in rice grains whereas high soil pH enhanced Cu bioaccumulation in rice grains. Previous studies^9,16^ have also reported that Cu accumulation in rice grains cannot be explained by soil Cu concentrations, while some soil properties could explain Cu bioaccumulation in rice grains. Compared to wheat and rape fields, the soil properties in the paddy fields seemed to influence metal bioaccumulation in rice grains more considerably. The reason for this could be soil systems in paddy fields are more complex^23^. Poor correlations between plant Pb and Fe concentrations and total or extractable soil Pb and Fe concentrations have been extensively reported by numerous researchers^8,9,12,16^. In the present study, there were also poor correlations between plant Pb and Fe concentrations and total or extractable soil Pb and Fe concentrations, despite the exploration of numerous soil properties and extraction methods.

## Conclusions

Soils from the paddy, wheat, and rape fields in the study area were all contaminated by Cd, and soils from the rape fields and the paddy fields exhibited Cu contamination. Spatial patterns of Cu, Zn, Pb, and Cd concentrations in soils were similar in the study area. The sites with high metal concentrations were mainly around mining sites, while the areas with lower soil metal concentrations were mainly far from the mining sites, indicating that metal contamination diminishes with an increase in distance from the mining sites. The PCA results also demonstrated that the Cu, Zn, Pb, Cd, and Mn contents were mainly influenced by mining activities. Soil pH or combinations of soil pH and total heavy metal concentrations in soils were the principal factors predicting NH_4_OAC and NH_4_NO_3_-extractable Zn, Pb, and Cd concentrations. Our results also indicated that mining activities had caused contamination of wheat and rice grains with Cd and Pb, and approximately 10% of the rice grain samples had Cd concentrations exceeding 1.0 mg/kg. The national government should raise awareness about the health risks posed by rice grains produced in the region. Furthermore, according to our results, rice and wheat grains exhibited stronger Cd accumulation than rape grains, and rape grains were safe, although soil Cd concentrations were generally high in the rape fields. Compared to rape and rice grains, metal accumulation in wheat grains could be better explained by soil metal concentrations. High soil pH and AK restricted Cd and Cu accumulation in wheat grains, respectively, and Cu and Zn accumulation in rice grains was also affected by soil pH and AK.

## Supplementary Information

Supplementary Information.

## Data Availability

All data generated or analyzed during this study are included in this published article [and its supplementary information files]. The raw data are available from the corresponding author on reasonable request.
