# Supplementary material for: Effects of soil properties on heavy metal bioavailability and accumulation in crop grains under different farmland use patterns
Source: Sci Rep. 2022 Jun 2;12:9211. doi: 10.1038/s41598-022-13140-1 (PMC9163331; doi:10.1038/s41598-022-13140-1)
Supplement: Supplementary file 1 — Supplementary Information. [file 41598_2022_13140_MOESM1_ESM.doc]

**Table S1 Correlation between soil properties and extractable metals in rape soils.**

| Metal(AI) | Extractant | pH | SOM | TN | TP | AP | AK |
| --- | --- | --- | --- | --- | --- | --- | --- |
| Cu | DTPA | 0.147(-0.297) | 0.400^*^(0.055) | 0.180(0.406^*^) | 0.326(-0.160) | -0.040(-0.214) | 0.085(-0.199) |
|  | EDTA | 0.262(0.033) | 0.428^**^(0.085) | 0.238(0.630^**^) | 0.367^*^(-0.110) | 0.016(-0.036) | 0.134(-0.107) |
|  | HCl | -0.005(-0.198) | 0.344^*^(0.155) | 0.296(0.287) | 0.257(0.082) | 0.006(-0.052) | -0.099(-0.257) |
|  | NH_4_OAC | 0.133(-0.087) | 0.306(-0.102) | 0.013(-0.056) | 0.333^*^(-0.062) | -0.022(-0.199) | 0.166(0.069) |
|  | NH_4_NO_3_ | -0.289(-0.491^**^) | 0.216(0.120) | -0.043(-0.076) | 0.223(0.146) | -0.054(-0.038) | 0.08(0.017) |
| Zn | DTPA | 0.282(-0.089) | 0.361^*^(0.148) | 0.335^*^(0.448^**^) | 0.562^**^(0.219) | 0.231(0.346^*^) | 0.347^*^(0.167) |
|  | EDTA | 0.458^**^ (0.305) | 0.388^*^(0.323) | 0.359*(0.480^**^) | 0.532^**^(0.307) | 0.218(0.294) | 0.410^*^(0.425^**^) |
|  | HCl | 0.468^**^(0.375*) | 0.333(0.238) | 0.460^**^(0.475^**^) | 0.570^**^(0.379*) | 0.265(0.425*) | 0.279(0.280) |
|  | NH_4_OAC | -0.234(-0.221) | 0.238(0.157) | 0.195(0.347^*^) | 0.378^*^(0.107) | 0.103(-0.027) | 0.082(0.075) |
|  | NH_4_NO_3_ | -0.746^**^(-0.747^**^) | 0.001(-0.015) | 0.062(0.032) | -0.037(0.130) | -0.099(0.093) | -0.135(-0.125) |
| Pb | DTPA | 0.205(-0.120) | 0.300(0.036) | 0.259(0.276) | 0.242(-0.299) | -0.208(-0.455^**^) | 0.123(-0.191) |
|  | EDTA | 0.382*(0.343*) | 0.353*(0.177) | 0.295(0.322) | 0.282(-0.165) | -0.157(-0.377*) | 0.234(0.104) |
|  | HCl | 0.072(-0.230) | 0.245(0.028) | 0.239(0.129) | 0.153(-0.297) | -0.319(-0.498^**^) | -0.103(-0.332) |
|  | NH_4_OAC | -0.400*(-0.476^**^) | 0.041(0.093) | 0.172(0.180) | -0.132(-0.102) | -0.426^**^(-0.306) | -0.148(-0.125) |
|  | NH_4_NO_3_ | -0.757^**^(-0.496^**^) | 0.055(0.086) | 0.02(-0.040) | 0.000(0.084) | -0.072(-0.029) | -0.145(-0.039) |
| Cd | DTPA | 0.381^*^(-0.438^**^) | 0.267(-0.261) | 0.271(0.016) | 0.316(-0.263) | -0.099(0.062) | 0.228(-0.215) |
|  | EDTA | 0.491^**^(-0.260) | 0.289(-0.271) | 0.292(0.073) | 0.333^*^(-0.231) | -0.067(0.133) | 0.271(-0.151) |
|  | HCl | 0.476^*^(-0.289) | 0.254(-0.211) | 0.377^*^(-0.053) | 0.360^*^(-0.151) | -0.026(0.125) | 0.138(-0.177) |
|  | NH_4_OAC | -0.005(-0.674^**^) | 0.152(-0.234) | 0.207(-0.047) | 0.083(-0.304) | -0.315(-0.039) | -0.014(-0.284) |
|  | NH_4_NO_3_ | -0.629^**^(-0.781^**^) | -0.002(-0.085) | 0.027(-0.109) | -0.059(0.014) | -0.269(0.033) | -0.185(-0.107) |
| Fe | DTPA | -0.707^**^(-0.722^**^) | 0.086(0.092) | 0.118(0.163) | -0.126(-0.109) | -0.079(-0.014) | -0.269(-0.256) |
|  | EDTA | -0.139(-0.178) | 0.333^*^(0.326) | 0.489^**^(0.479^**^) | 0.052(0.088) | 0.082(0.148) | -0.037(0.031) |
|  | HCl | -0.329(-0.245) | 0.152(0.204) | 0.107(0.211) | -0.120(-0.069) | -0.253(-0.070) | -0.324(-0.155) |
| Mn | DTPA | 0.560^**^(-0.771^**^) | -0.096(-0.190) | 0.131(0.120) | -0.270(-0.458^**^) | -0.220(-0.332^*^) | -0.023(-0.202) |
|  | EDTA | 0.244(0.05) | 0.186(0.044) | 0.278(0.413^*^) | -0.023(-0.362^*^) | -0.097(-0.290) | 0.248(-0.051) |
|  | HCl | 0.280(-0.045) | 0.355^*^(0.148) | 0.334(0.322) | 0.09(-0.287) | -0.150(-0.289) | 0.259(-0.109) |
|  | NH_4_OAC | -0.644^**^(-0.797^**^) | -0.173(-0.116) | 0.011(0.097) | -0.401^*^(-0.355^*^) | -0.391^*^(-0.272) | -0.332^*^(-0.148) |
|  | NH_4_NO_3_ | -0.706^**^(-0.821^**^) | -0.159(-0.048) | 0.007(0.068) | -0.278(-0.183) | -0.365^*^(-0.236) | -0.307(-0.098) |

Correlations refer to Log_10_-transformed data, except for pH (original data);AI: availability index;

**p* <0.05; ***p* <0.01.

**Table S2 Correlation between soil properties and extractable metals in wheat soils.**

| Metal(AI) | Extractant | pH | SOM | TN | TP | AP | AK |
| --- | --- | --- | --- | --- | --- | --- | --- |
| Cu | DTPA | 0.651^**^(0.278) | 0.509^**^(0.731^**^) | -0.139(0.301) | 0.541^**^(0.107) | 0.154(0.216) | 0.648^**^(0.319) |
|  | EDTA | 0.767^**^(0.517^**^) | 0.513^**^(0.517^**^) | -0.168(0.190) | 0.549^**^(0.144) | 0.177(0.296) | 0.682^**^(0.437^*^) |
|  | HCl | 0.696^**^(0.091) | 0.514^*^(0.694^**^) | -0.154(0.193) | 0.531^**^(0.013) | 0.169(0.291) | 0.650^**^(0.193) |
|  | NH_4_OAC | 0.603^**^(-0.093) | 0.371(0.017) | -0.091(0.320) | 0.567^**^(0.009) | 0.123(0.038) | 0.563^**^(-0.196) |
|  | NH_4_NO_3_ | 0.599^**^(0.158) | 0.130(-0.032) | -0.081(0.224) | 0.464^*^(-0.065) | 0.110(-0.024) | 0.361(-0.176) |
| Zn | DTPA | 0.406^*^(-0.043) | 0.374(0.208) | -0.184(0.104) | 0.481^*^(0.190) | 0.000(0.064) | 0.632^**^(0.163) |
|  | EDTA | 0.637^**^(0.558^**^) | 0.440^*^(0.458^*^) | -0.213(-0.092) | 0.551^**^(0.553^**^) | 0.082(0.235) | 0.727^**^(0.608^**^) |
|  | HCl | 0.632^**^(0.450^*^) | 0.429^*^(0.418^*^) | -0.272(-0.079) | 0.479^*^(0.346) | 0.083(0.264) | 0.725^**^(0.502^*^) |
|  | NH_4_OAC | -0.358(-0.419^*^) | -0.142(-0.145) | -0.170(0.162) | 0.070(-0.015) | -0.159(-0.463^*^) | 0.067(-0.260) |
|  | NH_4_NO_3_ | -0.857^**^(-0.725^**^) | -0.377(-0.294) | -0.099(0.183) | -0.243(-0.329) | -0.196(-0.160) | -0.424^*^(-0.567^**^) |
| Pb | DTPA | 0.265(0.219) | 0.514^**^(0.594^**^) | -0.075(0.075) | 0.189(0.012) | 0.041(0.127) | 0.497^*^(0.293) |
|  | EDTA | 0.480^*^(0.734^**^) | 0.524^**^(0.636^**^) | -0.131(-0.005) | 0.273(0.252) | 0.107(0.205) | 0.630^**^(0.557^**^) |
|  | HCl | 0.144(-0.094) | 0.458^*^(0.473^*^) | -0.119(0.045) | 0.069(-0.312) | 0.012(0.060) | 0.428^*^(0.041) |
|  | NH_4_OAC | -0.445^*^(-0.399^*^) | 0.196(0.113) | 0.125(0.005) | -0.395(-0.278) | -0.225(0.050) | -0.075(-0.130) |
|  | NH_4_NO_3_ | -0.865^**^(-0.708^**^) | -0.208(-0.154) | 0.157(0.238) | -0.538^**^(-0.543^**^) | -0.236(-0.128) | -0.588^**^ (-0.559^**^) |
| Cd | DTPA | 0.397^*^(-0.616^**^) | 0.510^**^(-0.128) | -.0104(0.116) | 0.359(-0.368) | -0.056(0.088) | 0.601^**^(-0.207) |
|  | EDTA | 0.514^**^(-0.219) | 0.550^**^(0.189) | -0.101(0.128) | 0.353(-0.258) | -0.021(0.123) | 0.648^**^(0.136) |
|  | HCl | 0.528^**^(-0.490^*^) | 0.551^**^(-0.049) | -0.157(0.063) | 0.396(-0.320) | 0.000(0.255) | 0.663^**^(-0.102) |
|  | NH_4_OAC | -0.132(-0.338) | 0.161(-0.187) | -0.062(0.071) | -0.012(-0.280) | -0.224(-0.180) | 0.183(-0.217) |
|  | NH_4_NO_3_ | -0.802^**^(-0.760^**^) | -0.313(-0.430^*^) | -0.112(0.112) | -0.256(-0.449^*^) | -0.186(-0.064) | -0.320(-0.496^*^) |
| Fe | DTPA | -0.802^**^(-0.778^**^) | -0.145(-0.176) | -0.013(0.102) | -0.262(-0.330) | 0.026(-0.005) | -0.583^**^(-0.656^**^) |
|  | EDTA | -0.014(-0.637^**^) | 0.339(-0.012) | -0.290(0.014) | 0.241(-0.239) | 0.271(0.113) | 0.034(-0.548^**^) |
|  | HCl | -0.538^**^(-0.675^**^) | -0.114(-0.133) | -0.140(0.067) | -0.237(-0.368) | 0.178(0.099) | -0.433^*^(-0.585^**^) |
| Mn | DTPA | -0.382(-0.753^**^) | -0.484^*^(-0.243) | 0.028(0.211) | -0.299(-0.589^**^) | -0.245(-0.230) | -0.368(-0.587^**^) |
|  | EDTA | 0.598^**^(0.665^**^) | -0.057(0.113) | -0.152(-0.168) | 0.244(0.196) | -0.044(0.043) | 0.342(0.365) |
|  | HCl | 0.683^**^(0.264) | 0.095(0.480^*^) | -0.062(0.198) | 0.215(-0.223) | -0.054(-0.041) | 0.383(0.147) |
|  | NH_4_OAC | -0.742^**^(-0.748^**^) | -0.442^*^(-0.129) | 0.150(0.250) | -0.483^*^(-0.574^**^) | -0.235(-0.203) | -0.587^**^(-0.566^**^) |
|  | NH_4_NO_3_ | -0.845^**^(-0.782^**^) | -0.501^*^(-0.171) | 0.098(0.226) | -0.474^*^(-0.558^**^) | -0.203(-0.206) | -0.624^**^(-0. 628^**^) |

Correlations refer to Log_10_-transformed data, except for pH (original data);AI: availability index;

**p* <0.05; ***p* <0.01.

**Table S3 Correlation between soil properties and extractable metals in paddy soils.**

| Metal(AI) | Extractant | pH | SOM | TN | TP | AP | AK |
| --- | --- | --- | --- | --- | --- | --- | --- |
| Cu | DTPA | 0.369(-0.416) | 0.664^**^(0.379) | 0.284(0.595^**^) | 0.451^*^(-0.453^*^) | -0.032(-0.424) | -0.120(-0.106) |
|  | EDTA | 0.488^*^(-0.161) | 0.695^**^(0.453^*^) | 0.278(0.633^**^) | 0.542^*^(-0.386) | 0.102(-0.172) | -0.096(0.028) |
|  | HCl | 0.291(-0.345) | 0.666^**^(0.356) | 0.415(0.535^*^) | 0.365(-0.481^*^) | -0.025(-0.621^*^) | 0.129(-0.190) |
|  | NH_4_OAC | 0.153(0.03) | 0.374(0.410) | 0.119(0.327) | 0.361(0.066) | -0.173(-0.328) | -0.240(-0.169) |
|  | NH_4_NO_3_ | 0.112(-0.288) | 0.430(0.160) | 0.131(0.189) | 0.378(-0.08) | -0.146(-0.352) | -0.011(0.030) |
| Zn | DTPA | 0.218(-0.257) | 0.589^**^(0.297) | 0.219(0.424) | 0.500^*^(-0.184) | 0.147(-0.151) | -0.114(-0.229) |
|  | EDTA | 0.374(0.095) | 0.619^**^(0.554^*^) | 0.202(0.460^*^) | 0.585^**^(0.224) | 0.256(0.163) | -0.064(-0.068) |
|  | HCl | 0.417(0.110) | 0.661^**^(0.498^*^) | 0.270(0.493^*^) | 0.631^**^(0.061) | 0.318(0.038) | -0.130(-0.328) |
|  | NH_4_OAC | -0.117(-0.242) | 0.218(0.379) | 0.094(0.406) | 0.040(-0.031) | -0.365(-0.236) | -0.074(-0.031) |
|  | NH_4_NO_3_ | -0.889^**^(-0.735^**^) | -0.078(0.100) | 0.247(0.359) | -0.477^*^(-0.360) | -0.388(-0.441) | 0.018(0.103) |
| Pb | DTPA | 0.112(-0.560^*^) | 0.383(-0.247) | -0.015(-0.048) | 0.543^*^(-0.369) | 0.192(-0.184) | 0.137(0.523^*^) |
|  | EDTA | 0.247(-0.253) | 0.498^*^(0.149) | 0.070(0.268) | 0.618^**^(-0.220) | 0.282(0.041) | 0.093(0.586^**^) |
|  | HCl | -0.156(-0.787^**^) | 0.399(-0.034) | 0.150(0.244) | 0.201(-0.665^**^) | -0.172(-0.605^**^) | 0.081(0.316) |
|  | NH_4_OAC | -0.674^**^(-0.863^**^) | 0.056(-0.125) | 0.133(0.155) | -0.239(-0.422) | -0.366(-0.386) | 0.237(0.413) |
|  | NH_4_NO_3_ | -0.917^**^(-0.776^**^) | -0.192(-0.333) | 0.108(0.028) | -0.408(-0.492^*^) | -0.420(-0.356) | 0.216(0.368) |
| Cd | DTPA | 0.268(-0.505^*^) | 0.591^**^(0.122) | 0.221(0.316) | 0.403(-0.459^*^) | 0.095(-0.283) | -0.026(0.062) |
|  | EDTA | 0.324(-0.189) | 0.622^**^(0.391) | 0.225(0.379) | 0.454^*^(-0.156) | 0.146(-0.082) | -0.028(0.031) |
|  | HCl | 0.359(-0.294) | 0.628^**^(0.222) | 0.260(0.345) | 0.467(-0.446) | 0.139(-0.353) | -0.038(-0.023) |
|  | NH_4_OAC | -0.064(-0.761^**^) | 0.511^*^(0.130) | 0.277(0.367) | 0.141(-0.470^*^) | -0.162(-0.469^*^) | 0.001(0.108) |
|  | NH_4_NO_3_ | -0.614^**^(-0.916^**^) | 0.161(-0.168) | 0.217(0.182) | -0.229(-0.521^*^) | -0.383(-0.467^*^) | 0.012(0.210) |
| Fe | DTPA | -0.769^**^(-0.822^**^) | -0.126(-0.231) | 0.257(0.261) | -0.630^**^(-0.653^**^) | -0.474^*^(-0.379) | -0.08(0.020) |
|  | EDTA | -0.361(-0.533^*^) | 0.159(0.056) | 0.326(0.416) | -0.305(-0.495^*^) | 0.034(-0.079) | 0.032(0.110) |
|  | HCl | -0.345(-0.486^*^) | 0.026(0.013) | 0.167(0.290) | -0.521^*^(-0.504^*^) | -0.673^**^(-0.505^*^) | -0.188(-0.212) |
| Mn | DTPA | -0.224(-0.777^**^) | 0.134(-0.097) | 0.038(0.189) | -0.118(-0.571^**^) | -0.299(-0.490^*^) | 0.037(0.056) |
|  | EDTA | 0.442(0.071) | 0.351(0.225) | 0.048(0.276) | 0.325(-0.202) | 0.167(-0.061) | 0.019(0.102) |
|  | HCl | 0.638^**^(0.209) | 0.538^*^(0.492^*^) | 0.157(0.482^*^) | 0.364(-0.258) | 0.049(-0.350) | -0.074(-0.139) |
|  | NH_4_OAC | -0.588^**^(-0.770^**^) | 0.034(-0.022) | 0.140(0.262) | -0.500^*^(-0.528^*^) | -0.699^**^(-0.456^*^) | 0.056(0.257) |
|  | NH_4_NO_3_ | -0.797^**^(-0.836^**^) | -0.1(-0.062) | 0.130(0.245) | -0.538^*^(-0.538^*^) | -0.598^*^(-0.481^*^) | 0.042(0.216) |

Correlations refer to Log_10_-transformed data, except for pH (original data);AI: availability index;

**p* <0.05; ***p* <0.01.

**Table S4 Correlation coefficients between the heavy metal concentrations in three grains (mg/kg, based on dry weight) and the total or extractable heavy metal concentrations in soil.**

| Metals |  | | | Rape grain | | | Wheat grain | | | | |
| --- | --- | --- | --- | --- | --- | --- | --- | --- | --- | --- | --- |
|  | Total | DTPA | EDTA | | NH_4_OAC | NH_4_NO_3_ | Total | DTPA | EDTA | NH_4_OAC | NH_4_NO_3_ |
| Cu | 0.532^**^ | 0.475^**^ | 0.490^**^ | | 0.341^*^ | 0.133 | 0.639^**^ | 0.605^**^ | 0.580^**^  0.428^*^  0.337  0.521^**^  0.092  -0.244 | 0.550^**^ | 0.488^*^ |
| Zn | 0.160 | 0.181 | 0.160 | | 0.103 | 0.204 | 0.524^**^ | 0.527^**^ |  | 0.534^**^ | 0.388 |
| Pb | 0.026 | 0.115 | 0.121 | | 0.012 | -0.101 | 0.205 | 0.330 |  | 0.268 | -0.037 |
| Cd | 0.348^*^ | 0.342^*^ | 0.367^*^ | | 0.175 | 0.022 | 0.380 | 0.574^**^ |  | 0.510^**^ | 0.537^**^ |
| Fe | 0.151 | -0.028 | 0.270 | | - | - | 0.335 | -0.278 |  | - | - |
| Mn | -0.030 | 0.553^**^ | 0.147 | | 0.482^**^ | 0.465^**^ | -0.126 | 0.578^**^ |  | 0.821^**^ | 0.808^**^ |

Soil total or extractable heavy metals concentrations and heavy metals concentrations of grains were Log_10_-transformed to ensure homogeneity of variances.**p* <0.05; ***p* <0.01.
